# The van Niel international prize for studies in bacterial systematics, awarded in 2026 to Jon Jongsik Chun

**DOI:** 10.1099/ijsem.0.007255

**Published:** 2026-07-20

**Authors:** Iain C. Sutcliffe, Edward R. B. Moore, Aharon Oren, On behalf of the International Committee on Systematics of Prokaryotes

**Affiliations:** 1 School of Geography and Natural Sciences, Northumbria University, Newcastle upon Tyne NE1 8ST, UK; 2 Department of Infectious Disease and Culture Collection University of Gothenburg (CCUG), Institute for Biomedicine, Sahlgrenska Academy, University of Gothenburg, Gothenburg, Sweden; 3 The Institute of Life Sciences, The Hebrew University of Jerusalem, The Edmond J. Safra Campus, 9190401 Jerusalem, Israel

## Abstract

The Senate of The University of Queensland, on the recommendation of the Executive Board of the International Committee on Systematics of Prokaryotes, is pleased to present the van Niel International Prize for Studies in Bacterial Systematics for the quadrennium 2020–2024 to Professor Jon Jongsik Chun in recognition of his contributions made to the field of bacterial systematics. The award, established in 1986 by Professor V.B.D. Skerman of the Department of Microbiology at the University of Queensland, honours the contribution of scholarship in the field of microbiology by Professor Cornelis Bernardus van Niel.

## Citation

Professor Jon Jongsik Chun studied in the Department of Microbiology at Seoul National University, Korea, before undertaking graduate research and obtaining his Ph.D. in microbial systematics in the laboratory of Professor Mike Goodfellow, with a thesis entitled, “Computer Assisted Classification and Identification of Actinomycetes” (awarded 1995), leading to the highly cited publication, ‘A Phylogenetic Analysis of the Genus Nocardia with 16S rRNA Gene Sequences’ [1]. His PhD was followed by post-doctoral research at the University of Maryland Biotechnology Institute and senior researcher position at the Korea Research Institute of Bioscience and Biotechnology. He has subsequently held various positions at the Seoul National University, where he has been full Professor in Bacteriology and Bioinformatics since 2009 and the founding director of the Bioinformatics Institute. Professor Chun is the founder and CEO of ChunLab Inc. since 2009 and, in 2019, launched CJ Bioscience Inc. as a platform for multiple bioinformatics tools for the microbiology community.

Professor Chun has co-authored more than 280 peer-reviewed publications, including more than eighty in the *International Journal of Systematic and Evolutionary Microbiology* (or its predecessor the *International Journal of Systematic Bacteriology*), with many highly influential publications on the application of sequence analysis methods in microbial systematics. These include landmark papers describing the widely used EzTaxon and EzBioCloud web-based tools that have provided high-quality platforms for 16S rRNA gene sequence and genomic analyses [2,5]. These studies have been complemented by major studies on the use of Average Nucleotide Identity for prokaryotic genome sequence analysis [6,7], including the development of the OrthoANI [8] and EzAAI tools [9]. His team have developed the UBCG pipeline for phylogenomic analysis, based on the ‘Up-to-date Bacterial Core Gene’ dataset [10,11], as well as establishing the UACG tool, based upon ‘Up-to-date Archaeal Core Gene’ dataset. He was the lead author on the influential ‘Proposed minimal standards for the use of genome data for the taxonomy of prokaryotes’ [12], whilst his 2014 paper with Fred Rainey introduced the term, ‘overall genome relatedness indices’ (OGRI) into microbial systematics vernacular [13]. In addition to his methodological innovations, he has contributed to the description of more than seventy bacterial species and seventeen genera, along with significant studies of the genomic epidemiology of *Vibrio cholerae*. In 2018, Professor Chun was awarded the Bergey’s Award and he was a trustee of Bergey’s Manual Trust from 2009 to 2016. More recently, Professor Chun has focused his research on the diversity and ecological structure of gut microbiomes.

The above citation accompanies the prize. The International Committee on Systematics of Prokaryotes congratulates Professor Chun on being awarded the prize in recognition of his outstanding contributions to prokaryote systematics. As part of the legacy of Professor Skerman, the International Committee on Systematics of Prokaryotes recommends to the officials at the School of Chemistry and Molecular Biosciences of the University of Queensland a candidate who has excelled in development and studies in fields of microbial systematics and taxonomy to be awarded the prize. A history of the van Niel Prize, listing previous recipients, is given in Appendix 12 of the International Code of Nomenclature of Prokaryotes [14,15].

Citation prepared by The University of Queensland and submitted on their behalf and that of the International Committee on Systematics of Prokaryotes by I.C. Sutcliffe, E.R.B. Moore, and A. Oren.

## Abbreviations

OGRI: Overall Genome Relatedness Indices
UACG: Up-to-date Archaeal Core Genes
UBCG: Up-to-date Bacterial Core Genes
